# Polyanionic Lattice Modifications Leading to High‐Entropy Sodium Ion Conductors: Mathematical Solution of Accessible Compositions

**DOI:** 10.1002/cphc.202000566

**Published:** 2020-08-26

**Authors:** Frank Tietz, Carsten Fronia

**Affiliations:** ^1^ Institut für Energie- und Klimaforschung, IEK-1: Materials Synthesis and Processing Institut für Energie- und Klimaforschung, IEK-12: Helmholtz-Institute Münster Forschungszentrum Jülich GmbH 52425 Jülich Germany; ^2^ Pi-Counter Unternehmensberatung Carsten Fronia Bartold-Knaust-Str. 2 30459 Hannover Germany

**Keywords:** high-entropy materials, NaSICON, numerical analysis, polyanionic substitutions

## Abstract

Sodium zirconium double phosphate NaZr_2_(PO_4_)_3_ can be used as a starting point for investigations of high‐entropy materials. Apart from the frequently used approach of partial substitution with four or more different transition metal cations, this class of materials also allows multiple substitutions of the phosphate groups. Herein modifications of the polyanionic lattice are considered and high‐entropy compositions are numerically determined with up to eight elements on the central tetrahedral lattice site of the so‐called NaSICON structure. For this study, the chemical formula was fixed as Na_3_Zr_2_(EO_4_)_3_ with E=B, Al, Si, P, As, Sb, S, Se and Te. The number of compositions increases exponentially with the increasing number of elements involved and with decreasing equal step size for each element. The maximum number of 237258 compositions is found for Na_3_Zr_2_([B,Al,Si,P,As,Sb,S,Se]O_4_)_3_ with a step size of 0.1 mol/formula unit. Of this compositional landscape, 143744 compositions fulfil the definitions of high‐entropy materials. The highest entropy factor of ΔS_config_/*R*=‐2.0405 is attributed to the compositions Na_3_Zr_2_(B_0.5_Al_0.6_Si_0.4_P_0.3_As_0.3_Sb_0.3_S_0.3_Se_0.3_)O_12_ and Na_3_Zr_2_(B_0.6_Al_0.5_Si_0.4_P_0.3_As_0.3_Sb_0.3_S_0.3_Se_0.3_)O_12_.

## Graphical abstract

**How many compositions exist** for Na_3_Zr_2_(XO_4_)_3_ with E=B, Al, Si, P, As, Sb, S, Se? With a step size of 0.1 mol, 237258 compositions can be determined. 143744 of these are high‐entropy compositions and 3331 of these do not contain phosphorus. More mathematical facts can be explored herein.

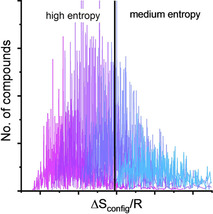

## 1 Introduction

In recent years, the study of high‐entropy materials has attracted increasing interest with the aim of finding materials with improved properties. In the case of high‐entropy alloys, a wide variety of alloys with improved mechanical, magnetic and sometimes electrical properties have been observed, see e. g.^1^, ^2^ and references therein. In the case of high‐entropy oxides, the number of examples is much less although the basic concept dates back to the 1960s.^3^ To our knowledge, only the cationic sites in oxides with different crystal structures have been used as yet to induce high disorder. The use of equimolar amounts of various transition metal ions in compounds with rock salt structure is the most generic example^4^, ^5^ and has shown unique electrochemical properties when applied as the cathode^4^, ^6^ or anode^7^ in lithium‐ion batteries. Obviously, Li^+^ ions can be easily incorporated into and extracted from the strongly disordered rock salt structure, which is supported by the very high ionic conductivity of up to 1 mS/cm for (Mg,Co,Ni,Cu,Zn)_0.67_Li_0.33_O.^8^ Apart from further studies on materials with rock salt structure,^9^ high‐entropy oxides with spinel,^10^ fluorite,^11^ bixbyite,^12^ perovskite^13^ and magnetoplumbite structure^14^ have been created and investigated. Further examples of high‐entropy oxides can be found elsewhere.^15^ For quinary rock salt compositions, Anand et al.^16^ calculated the compositional configurations on the basis of a supercell with 2000 atoms considering interatomic potentials and molecular dynamics simulations resulting in the thermodynamic quantities for the evaluation of stable configurations.

The increasing number of publications on high‐entropy oxides also led to the idea of creating high‐entropy NaSICON (Na^+^ superionic conductor) materials^17^ because this class of materials is known for the flexible incorporation of many elements. To our knowledge, the high‐entropy oxides investigated so far have been based on multiple elements on cationic sites with equal fractions of the cations involved in order to maximize the entropy.^4^, ^5^, ^6^, ^7^, ^8^, ^9^, ^10^, ^11^, ^12^, ^13^, ^14^, ^15^ On the one hand, this approach can also be applied to NaSICON materials, because a large variety of cations can be incorporated.^18^ On the other hand, however, this class of materials also offers the opportunity to induce high disorder in the polyanionic lattice in terms of variable site occupancy with many different central tetrahedral ions, which is not possible for the oxides mentioned above. This attempt can offer a new path to fast Na^+^ ion conductors due to the permanently changing next‐nearest neighbors of the Na^+^ ions inducing modulated bond strengths along the conduction paths. A systematic variation of the central tetrahedral ions in the polyanionic sub‐lattice can also elucidate fundamental details of ion transport in this class of materials. Finally, as in the case of perovskites in which the multiple substitution of two different cation sites was already investigated,^13^ the cationic lattice offers the opportunity to further increase the entropy. In this report, however, we restrict our calculations to the polyanionic sub‐lattice.

## 2 General Considerations Regarding Polyanionic Substitutions

Here we present a first approach for high‐entropy oxides by multiple substitutions in the polyanionic lattice of NaSICON, which is isostructural with kosnarite (KZr_2_(PO_4_)_3_).^19^ Starting from the generic formula NaZr_2_(PO_4_)_3_, the solid solution Na_1+x_Zr_2_(SiO_4_)_x_(PO_4_)_3‐x_ ends with Na_4_Zr_2_(SiO_4_)_3_ and compositions with 2<x<2.5 have shown the highest ionic conductivity of all NaSICON materials.^17^, ^20^ The variation of the phosphate groups can theoretically be extended by many polyanions starting from (LiO_4_)^7−^ to (WO_4_)^2−^ with central tetrahedral elements such as Li, Mg, Ti, V, W, Fe, Cr, Mn, Zn, B, Al, Ga, Si, Ge, Sn, P, As, Sb, S, Se, similar to the wide chemical variability of garnets^21^ or glaserites.^22^ For the sake of simplicity, we restrict the selection of polyanions to those which a) do not belong to transition elements excluding compositions that might act as electrode materials instead of solid electrolytes, b) are fairly cheap and abundant leading to the exclusion of polyanions such as (GaO_4_)^5−^and (GeO_4_)^4−^ and c) have unrealistically large ionic radii or unusual valencies, which presumably do not fit into the NaSICON structure. As a result, the only polyanions considered here are listed in Table 1. Even this limited number of elements contains some uncertainties with respect to applicability as will be discussed further below.

**Table 1:** Polyanions considered for high‐entropy NaSICON materials.

Element (E)	Cation valency	Polyanion	Ionic radius (*r_E_*) in tetrahedral coordination^23^ [Å]
B	+3	(BO_4_)^5−^	0.11
Al	+3	(AlO_4_)^5−^	0.39
Si	+4	(SiO_4_)^4−^	0.26
P	+5	(PO_4_)^3−^	0.17
As	+5	(AsO_4_)^3−^	0.335
Sb	+5	(SbO_4_)^3−^	(0.49)^[a]^
S	+6	(SO_4_)^2−^	0.12
Se	+6	(SeO_4_)^2−^	0.28
Te	+6	(TeO_4_)^2−^	0.43

**Figure 1:**
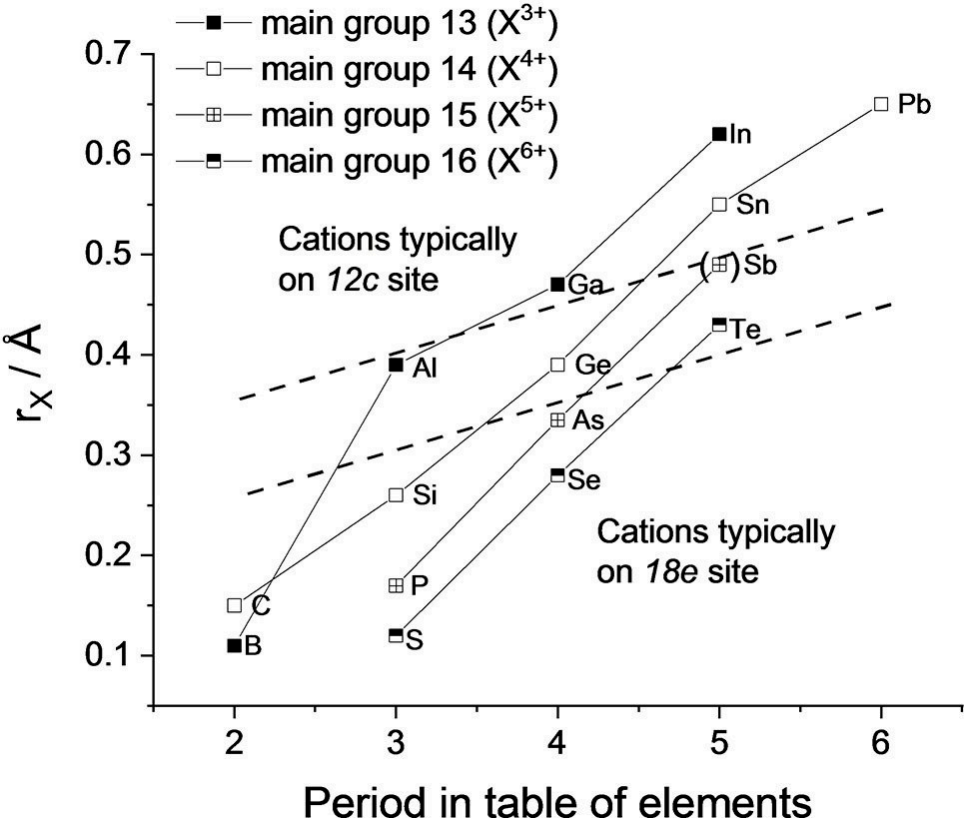
Ionic radii of main group cations in tetrahedral coordination.^23^ The value of Sb^5+^ was extrapolated according to the slope of the neighboring tetravalent and hexavalent cations. The cations between the two dashed lines can occupy both lattice sites (Al, Ge) or may require experimental confirmation (Sb, Te).

[a] No value given in Ref. [23]; extrapolated value from Figure 1.

Although a vast amount of publications is available for cation substitutions of NaSICON materials, the number of modifications on the anionic lattice is rather low. Apart from the well‐investigated substitution of P^5+^ by Si^4+^, the isovalent substitution with As^5+^ is also reported for AZr_2_P_3‐x_As_x_O_12_ with A=Li, Na, K, Rb, Cs.^24^ However, to our knowledge no composition has yet been reported in which P^5+^ has been replaced by Sb^5+^. As can be seen in Figure 1, this seems to be reasonable because Sb^5+^ should have a similar ionic radius to Ga^3+^ in tetrahedral coordination. These cations are known as substitution elements for Zr^4+^ on the octahedrally coordinated *12c* site.^25^ Similarly, Al^3+^ and Ge^4+^ have nearly the same ionic radius (see Figure 1) and are also known as substituents of Zr^4+^.^25^, ^26^ However, in the case of Ge^4+^ also the substitution of P^5+^ on the *18e* site is reported, but with limited solubility.^27^ According to these facts, two tentative dashed lines are inserted in Figure 1 to distinguish between cations occupying *12c* and *18e* sites. The elements between the two dashed lines can occupy both sites or are assumed to do so. The slope of these lines is rather arbitrary, because it remains to be experimentally clarified whether Al^3+^, Sb^5+^ and Te^6+^ can partially occupy *18e* sites.

In the case of hexavalent cations (S, Se), only compositions with modified cationic lattices are known, but not with Zr^4+^ or any other tetravalent cation as the single transition metal cation on the *12c* site. However, Savinykh et al.^28^ investigated various phosphate‐sulfates with simultaneous substitutions of Zr^4+^ with di‐ and trivalent cations. In the case of sulfates, the most frequently investigated NaSICON material is Fe_2_(SO_4_)_3_ as the cathode material in sodium‐ion batteries.^29^ In addition, Slater and Greaves^30^ investigated a large number of materials with the general composition A_x_M^II^_x_M^III^_2‐x_(SO_4_)_3‐y_(SeO_4_)_y_, justifying the inclusion of Se in the following considerations. Te^6+^ has a similar ionic radius to Ge^4+^ in tetrahedral coordination and can be considered as a substituent for *12c* and *18e* sites as discussed above.

For the trivalent central ions (B, Al), there is no information on targeted substitutions in the polyanionic lattice. However, Maldonado‐Manso et al. showed by NMR measurements that Al^3+^ can simultaneously occupy *12c* and *18e* sites in Li_1+x_Al_x_Ge_2‐x_(PO_4_)_3_.^31^ In the case of B^3+^, the use of high amounts of borates as a sintering aid^32^ may imply limited interaction with the crystalline material and potentially minor substitutions in the nominal ceramics. However, usually small amounts of borates are added to stoichiometric compounds to influence grain boundary chemistry and sintering properties, but are not intended to replace other polyanions. Two other reports used B_2_O_3_ as an additive in the synthesis of Li_1+x_Al_x_Ti_2‐x_(PO_4_)_3_ from the melt.^33^ In both cases, the attempt was made to substitute Al^3+^ with B^3+^, which is a misconception of the experiments according to Figure 1. Although it was stated that B^3+^ is incorporated into the NaSICON structure, it is not unambiguously clear on which position in the crystal lattice and in what amount. Therefore, again, further experimental or computational work has to define the solubility limits of (BO_4_)^5−^ and (AlO_4_)^5−^ in the NaSICON structure before the expected decomposition or glass formation will occur.

For the following mathematical approach, however, these two polyanions as well as (SbO_4_)^3−^ and (TeO_4_)^2−^ are included in the calculations to elucidate the determination of the number of compositions that can be expected by implementing up to eight different polyanions instead of the exclusive presence of phosphate groups. In forthcoming contributions, we will refine the calculation according to the solubility limit of the uncertain cations. If some elements have to be excluded for chemical reasons, calculations are also provided with decreased numbers of cations involved, down to five elements on the phosphorus site.

## 3 Chemical and Mathematical Boundary Conditions

In order to determine the compositional space considering the selected elements that can occupy the central tetrahedral site in the NaSICON structure, the possible permutations were numerically counted with specific boundary conditions:

1. The compositions were fixed in the cationic lattice as the composition Na_3_Zr_2_(EO_4_)_3_.
2. All elements in Table 1 are allowed with molar contents between 0 and 3 moles per formula unit.
3. The number of elements (*N*) was varied between 5<*N*<8 and for *N*=8 two different calculations were carried out using either Sb^5+^ or Te^6+^.
4. According to boundary condition 1, the molar sum (X) of all E is always 3 (Σ m_i_=3), i. e. no vacant *18e* sites are allowed.
5. According to boundary condition 1, for charge neutrality the sum of positive charges (q^+^) is always 13 (see Table 1), i. e. no charge compensation by additional sodium ions or oxygen vacancies is allowed.

Parameters for the numerical counting are the lower and upper limit of the molar amounts of each element and the step size in which the stoichiometry of each element is changed. Therefore

6. In each numerical solution the step size, S’, was kept constant for all elements. Here step sizes of S’=0.1, 0.2, 0.25 and 0.5 mol per formula unit were chosen.

The sequence of numerical counting is illustrated in Figure S1 in the supporting information. The programme was written with the SAS software package (SAS Institute Inc, Cary, NC, USA; version 9.4 (TS1 M6)).

## 4 Results and Discussion

### 4.1 Total Number of Possible Compositions

Neglecting the boundary conditions above, the total number of permutations can be calculated with the formula N^X/S’+1^. For N=8, X=3 and S’=0.1 this permutation results in about 10^28^ solutions. Applying the boundary conditions, only a small fraction of results remain as chemically reasonable solutions. The extraction of the meaningful results have been filtered with the computed programme as shown in Figure S1.

Table 2 gives a statistical overview of the achievable number of compositions for S’=0.1 mol depending on the number and type of E irrespective of any entropy considerations. The first result worth noting is the total sum of compositions for *N*=8: whereas the presence of three pentavalent cations (P, As, Sb) gives 237258 compositions, the presence of three hexavalent cations (S, Se, Te) reduces the number of compositions by more than 50000 due to the different constellations for fulfilling charge neutrality. The decrease of *N* also significantly (exponentially) decreases the total number of compositions. It is also interesting to note that for *N*=6 or 5 the number of compositions can vary strongly depending on the selected cations. Only two examples of both *N* are listed in Table 2 to demonstrate the impact of this selection.

**Table 2:** Statistical overview of the total number of compositions and the involvement of each element for the counts with S’=0.1 mol. In the first line of each case the absolute numbers of compositions are given in which the corresponding element appears, the second line corresponds to the percentage of appearances and the third line shows the maximum molar amount (*m*_*i,max*_) that can be obtained for each element.

E	Total	B	Al	Si	P	As	Sb	S	Se	Te
B, Al, Si, P, As, Sb, S, Se	237258	213108	213108	202020	191163	191163	191163	179347	179347	–
[%]		89.82	89.82	85.15	80.57	80.57	80.57	75.59	75.59	–
*m*_i,max_		1.6	1.6	2.5	2.0	2.0	2.0	1.3	1.3	–
B, Al, Si, P, As, S, Se, Te	184422	167412	167412	158214	148260	148260	–	138327	138327	138327
[%]		90.78	90.78	85.79	80.39	80.39	–	75.01	75.01	75.01
*m*_*i,max*_		1.6	1.6	2.5	2.0	2.0	–	1.3	1.3	1.3
B, Al, Si, P, As, S, Se	46095	41412	41412	40131	38696	38696	–	37099	37099	–
[%]		89.84	89.84	87.06	83.95	83.95	–	80.48	80.48	–
*m*_*i,max*_		1.6	1.6	2.5	2.0	2.0	–	1.3	1.3	–
B, Al, Si, P, As, S	8996	7938	7938	7904	7942	7942	–	7830	–	–
[%]		88.24	88.24	87.86	88.28	88.28	–	87.04	–	–
*m*_*i,max*_		1.6	1.6	2.5	2.0	2.0	–	1.3	–	–
B, Si, P, As, S, Se	4683	4592	–	4263	3941	3941	–	3625	3625	–
[%]		98.06	–	91.03	84.16	84.16	–	77.41	77.41	–
*m*_*i,max*_		1.6	–	2.5	2.0	2.0	–	1.3	1.3	–
B, Si, P, As, S	1058	1022	–	974	934	934	–	882	–	–
[%]		96.60	–	92.06	88.28	88.28	–	83.36	–	–
*m*_*i,max*_		1.6	–	2.5	2.0	2.0	–	1.3	–	–
Si, P, As, S, Se	91	–	–	91	70	70	–	55	55	–
[%]		–	–	100	76.92	76.92	–	60.44	60.44	–
*m*_*i,max*_		–	–	2.5	1.0	1.0	–	0.5	0.5	–

**Figure 2:**
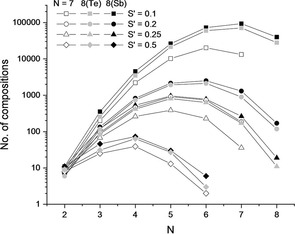
Dependence of resulting compositions on step size and number of involved elements for three specific cases in which 7 or 8 elements are allowed (see first three cases in Table 2).

Another factor strongly decreasing the number of possible compositions is the step size for varying the compositional changes (Figure 2). Considering the case with *N*=6, the number of solutions varies from 2 (S’=0.5 mol) to 20235 (S’=0.1 mol) when a total of 7 elements are allowed (open symbols in Figure 2). The figure also shows that the maximum variability in compositions is observed for N‐1, N‐2, N‐3 and N‐2 for step sizes 0.1, 0.2, 0.25 and 0.5 mol, respectively.

**Figure 3:**
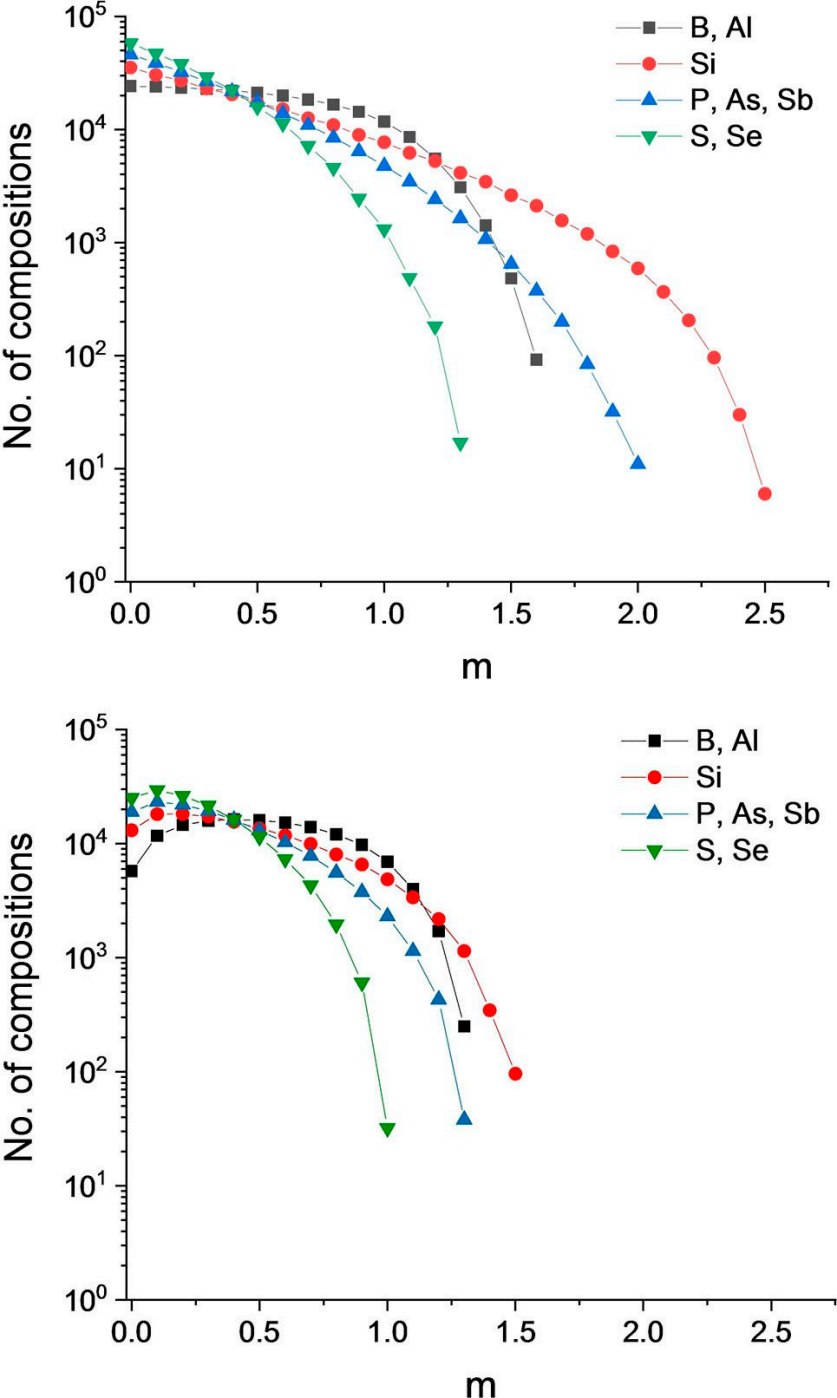
Distribution of molar amounts for the various cations in the case of *N*=8 with Sb and S’=0.1 mol (see first line in Table 2). Top: total set of compositions, bottom: high‐entropy compositions.

The molar amount of the elements varies from nearly equimolar distributions to maximum achievable amounts as listed in Table 2. The distribution of *m_i_* varies among the elements of different charge and an example (*N*=8 with Sb and S’=0.1 mol) is shown in Figure 3 (top).

Finally, it is worth mentioning that a mathematical curiosity was observed when only one tri‐, tetra‐ and pentavalent cation was used for the numerical determination of possible compositions. With this set of three elements, 11, 101 and 1001 solutions were found for step sizes of 0.1, 0.01 and 0.001 mol, respectively.

### 4.2 Number of Compositions with *N*≥5

The first definition that has to be fulfilled by a high‐entropy material is the presence of at least five elements either as major metal elements in alloys or as substituents on one single site in an inorganic material. Using this definition, maximum entropy can be achieved in a system when the multiple elements appear in an equimolar ratio. This definition can be used here as a first filter for the high numbers of possible compositions excluding all solutions with *N*<5. However, comparing the values in Table 2 and Table S1 in the supporting information, the numbers are still large and show that solutions with only 2, 3, or 4 elements are rather rare compared to the values with 5 or more elements.

### 4.3 Number of High‐Entropy Compositions

The second requirement for a high‐entropy material is based on statistical thermodynamics. A threshold of configurational entropy of 1.609 *R* is defined as a minimum value for the randomly distributed cations or elements in a solid solution. This value results from Equation (1):

(1) $${\Delta S}_{config\;}=\;-R\sum_{i}^{n}x_{i}ln{\;x}_{i}$$

where *R* is the gas constant and *x_i_* are the molar fractions. Table S2 gives the results for 1<*n*<9. When *n*=5 the above‐mentioned threshold for high‐entropy materials is obtained.

For an inorganic salt, Equation 1 can be separated into a cationic and an anionic part, but since the solid solution is usually only related to one of the sublattices, only this part also defines Δ*S_config_*. Hence, for the NaSICON materials considered here, Equation (1) can be rewritten as Equation (2):

(2) $${-\Delta S}_{config\;}/R=\sum_{i}^{N}\frac{m_{i}}{3}\ln\frac{{\;m}_{i}}{3}$$

to normalize the molar amounts and use them as molar fractions. The resulting sum, the entropy factor, was calculated for all compositions and in order to fulfil the second definition, the values have to be given with a precision of 5 digits.

With this second definition, the number of compositions is greatly reduced (Table 3). In particular the compositions with high molar amounts of one element are eliminated, as can also be seen in Figure 3 (bottom). Remarkably, none of the variations with *N*=5 result in compositions with Δ*S_config_/R*<−1.6094 and the highest value was found to be as low as −1.6039. Also for *N*=6 and E=B, Si, P, As, S, Se there are only a limited number of 264 compositions that surpass the threshold. This number is reduced to 18 and 7 compositions for S’=0.2 and 0.25 mol, respectively. No composition can be identified with S’=0.5 mol.

**Table 3:** Statistical overview of the number of compositions with *N*≥5 and Δ*S_config_/R*≤‐1.6094 and the involvement of each element for counts with S’=0.1 mol. In the first line of each case the absolute numbers of compositions are given in which the corresponding element appears, the second line corresponds to the percentage of appearances and the third line shows the maximum molar amount (*m*_*i,max*_) that can be obtained for each element.

E	Total	B	Al	Si	P	As	Sb	S	Se	Te
B, Al, Si, P, As, Sb, S, Se	143744	138017	138017	130706	124821	124821	124821	118642	118642	–
[%]		96.02	96.02	90.93	86.84	86.84	86.84	82.54	82.54	–
m_i,max_		1.3	1.3	1.5	1.3	1.3	1.3	1.0	1.0	–
B, Al, Si, P, As, S, Se, Te	104584	102047	102047	96036	91114	91127	–	85661	85659	85676
[%]		97.57	97.57	91.83	87.12	87.13	–	81.91	81.90	81.92
m_i,max_		1.4	1.4	1.6	1.4	1.4	–	1.0	1.0	1.0
B, Al, Si, P, As, S, Se	18908	18644	18644	17986	17545	17543	–	17147	17145	–
[%]		98.60	98.60	95.12	92.79	92.78	–	90.69	90.68	–
m_i,max_		1.3	1.3	1.4	1.2	1.3	–	1.0	1.0	–
B, Al, Si, P, As, S	1763	1763	1763	1763	1763	1763	–	1763	–	–
[%]		100	100	100	100	100	–	100	–	–
m_i,max_		1.0	1.0	1.2	1.1	1.3	–	1.0	–	–
B, Si, P, As, S, Se	264	3768	–	3581	3391	3391	–	3184	3184	–
[%]		100	–	100	100	100	–	100	100	–
m_i,max_		1.2	–	1.1	0.8	0.9	–	0.5	0.6	–

**Figure 4:**
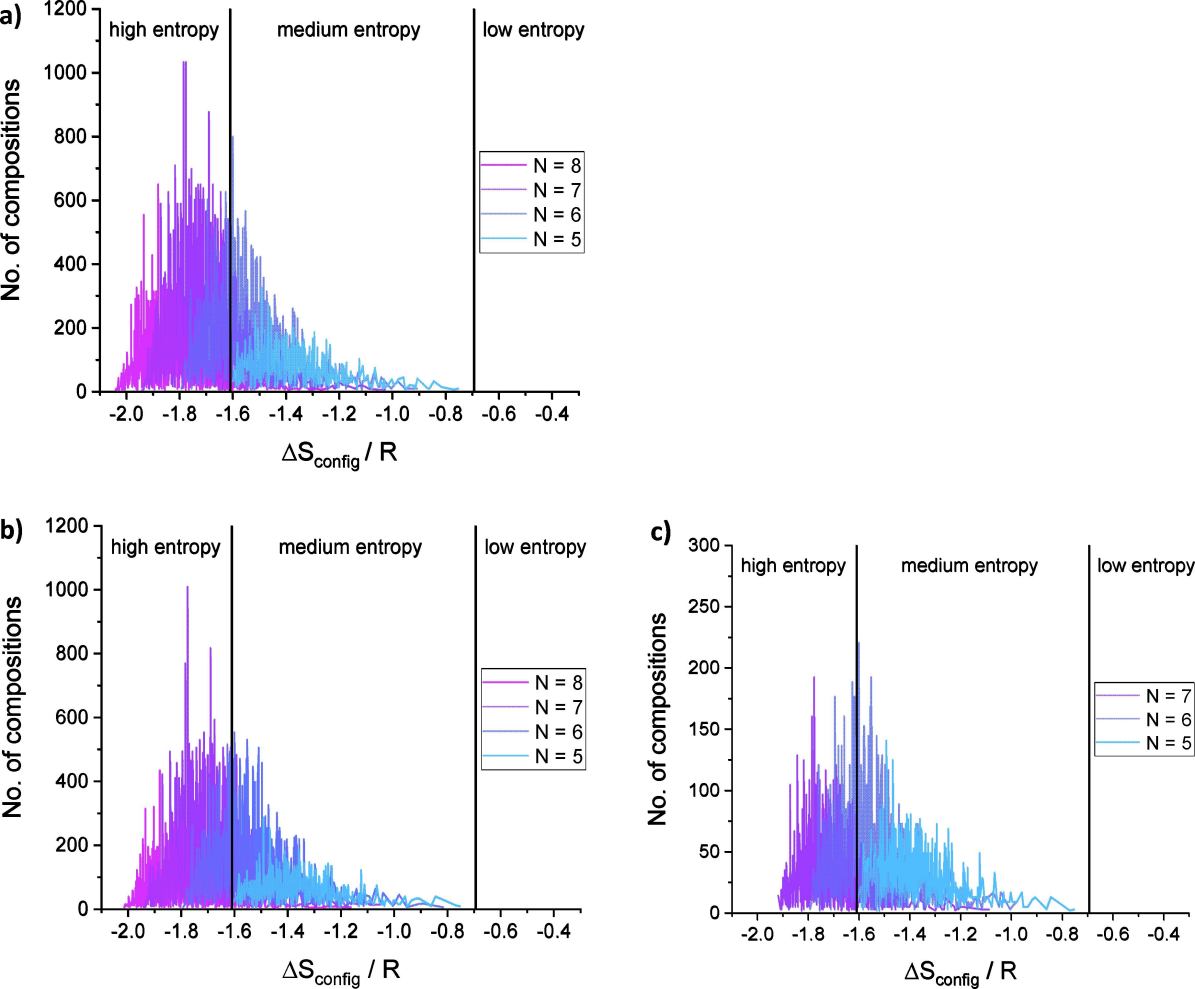
Distribution of entropy factors Δ*S_config_/R* for the three cases *N=*8 with Sb (a), *N=*8 with Te (b) and *N=*7 (c) as listed in Table 3. In each of the three cases, the solutions are separated into contributions with 5<N<8 elements. Additional colors appear due to the partial transparency in order to better visualize the individual contributions.

Figure 4 displays the distribution of entropy factors for the first three cases in Table 3. Here also the values for *N*=5 are included even though none of these compositions can be regarded as high‐entropy NaSICONs. Table S3 summarizes the statistical data of entropy factors for all compositions as well as for the high‐entropy NaSICONs, both valid for S’=0.1 mol.

In addition to the more general evaluation of the numerical results, Table 4 shows individual compositions with the highest entropy per elemental configuration. As can be seen here very clearly, the most homogeneous distribution of molar amounts results in the highest entropy factor of Δ*S_config_/R*=‐2.0405. Comparing the results with *N*=8, the participation of three pentavalent cations is slightly more beneficial for the entropy than the presence of three hexavalent cations due to the unequal molar amounts among these three cations. From this table it is also evident that Δ*S_config_/R* increases with decreasing *N* and that solutions with *N*=5 do not belong to the category of high‐entropy materials. Additionally, the number of solutions decreases due to the increasing limitations for permutations. Therefore, the solutions for *N*=6 with equimolar amounts of all cations – and therefore with constant Δ*S_config_/R* – decrease from six to only one single solution when 8 and only 6 cations are involved, respectively.

**Table 4:** Examples of NaSICON compositions according to Na_3_Zr_2_(EO_4_)_3_ with the highest entropy per elemental configuration and the number of equivalent solutions.

N	B	Al	Si	P	As	Sb	S	Se	Te	ΔS_config_/R	Solutions
E=B, Al, Si, P, As, Sb, S, Se											
5	0.6	0.7	0	0	0.5	0.6	0.6	0	–	−1.6039	32
6	0.5	0.5	0.5	0	0.5	0.5	0.5	0	–	−1.7918	6
7	0.5	0.5	0.4	0.4	0.4	0.4	0	0.4	–	−1.9405	2
8	0.5	0.6	0.4	0.3	0.3	0.3	0.3	0.3	–	−2.0405	2
E=B, Al, Si, P, As, S, Se, Te											
5	0.6	0.6	0.7	0	0	–	0.5	0	0.6	−1.6039	18
6	0.5	0.5	0.5	0.5	0.5	–	0	0	0.5	−1.7918	3
7	0.5	0.6	0.4	0.5	0.4	–	0	0.2	0.4	−1.9170	39
8	0.6	0.6	0.4	0.3	0.3	–	0.2	0.2	0.4	−2.0140	3
E=B, Al, Si, P, As, S, Se											
5	0.6	0.6	0.7	0	0	–	0.5	0.6	–	−1.6039	10
6	0.5	0.5	0.5	0.5	0.5	–	0	0.5	–	−1.7918	2
7	0.5	0.6	0.5	0.3	0.4	–	0.3	0.4	–	−1.9170	13
E=B, Al, Si, P, As, S											
5	0.7	0.6	0	0.5	0.6	–	0.6	–	–	−1.6039	4
6	0.5	0.5	0.5	0.5	0.5	–	0.5	–	–	−1.7918	1
E=B, Si, P, As, S, Se											
5	0.9	–	0.6	0.5	0.6	–	0	0.4	–	−1.5722	4
6	1	–	0.6	0.4	0.4	–	0.3	0.3	–	−1.6859	1

On the one hand the presented method of numerical counting delivers all possible and chemically reasonable compositions and on the other hand it offers the filtering of compounds in terms of composition, entropy or any other property when the output list (see Figure S1) is used as an input file and combined with first‐principles calculations or other atomistic calculation methods.^34^ In this way also the thermodynamic stability of the compositions can be evaluated. This approach has similarities with computational screening of new materials based on high‐throughput and machine‐learning techniques followed by density functional theory (DFT) calculations.^34^, ^35^ In these cases, however, the compositional space is narrowed by heuristic or probabilistic models as machine‐learning step whereas here the chemical intuition was used. Another difference of both approaches is the fact that, to the best of our knowledge, the computational search for new materials is restricted yet to quaternary systems, but here up to eight different elements are considered not including the cationic sub‐lattice. Whatever method is used to filter new compounds, the listed candidates have to be verified for thermodynamic stability using DFT calculations.^16^, 35b Furthermore, the impact of substitutions on the ionic transport properties is usually also calculated with DFT methods. However, in comparison to single^36^ or double35c ion substitutions, the multiple substitutions discussed here may cause significant computational effort for the atomistic calculations due to the definition of very large supercells and a huge number of variable site occupancies of randomly distributed ions in the tetrahedral sites. For this challenging computation a machine‐learning technique would also be highly valuable.

## 5 Conclusion

For the general formula Na_3_Zr_2_(EO_4_)_3_ with E=B, Al, Si, P, As, Sb, S, Se, Te, the number of possible compositions as well as the high‐entropy compositions were determined numerically with up to eight elements on the central tetrahedral lattice site of the NaSICON structure. The number of compositions increases exponentially with increasing number of elements involved and with decreasing step size. The maximum number of 237258 compositions was found for Na_3_Zr_2_([B,Al,Si,P,As,Sb,S,Se]O_4_)_3_ with S’=0.1 mol, whereas 143744 of these solutions, i. e. 60.6 %, are high‐entropy compositions. The compositions with the highest entropy factor of Δ*S_config_/R*=‐2.0405 are Na_3_Zr_2_(B_0.5_Al_0.6_Si_0.4_P_0.3_As_0.3_Sb_0.3_S_0.3_Se_0.3_)O_12_ and Na_3_Zr_2_(B_0.6_Al_0.5_Si_0.4_P_0.3_As_0.3_Sb_0.3_S_0.3_Se_0.3_)O_12_. Experimental and/or computational work – by analogy with^16^, ^35^ – is necessary to verify the solubility range of several elements and the existence of solid solutions with so many elements in the polyanionic lattice.

## Conflict of interest

The authors declare no conflict of interest.

## Supporting information

As a service to our authors and readers, this journal provides supporting information supplied by the authors. Such materials are peer reviewed and may be re‐organized for online delivery, but are not copy‐edited or typeset. Technical support issues arising from supporting information (other than missing files) should be addressed to the authors.

Supplementary

## References

[1] Y. Zhang , High-Entropy Materials, Springer Nature Singapore Pte Ltd., 2019.

[2] D. B. Miracle , O. N. Senkov , Acta Mater. 2017, 122, 448–511.

[3] A. Navrotsky , O. J. Kleppa , J. Inorg. Nucl. Chem. 1967, 29, 2701–2714.

[4] C. M. Rost , E. Sachet , T. Borman , A. Moballegh , E. C. Dickey , D. Hou , J. L. Jones , S. Curtarolo , J.-P. Maria , Nat. Commun. 2015, 6, 8485. 26415623 10.1038/ncomms9485 PMC4598836

[5] A. Sarkar , L. Velasco , D. Wang , Q. Wang , G. Talasila , L. de Biasi , C. Kübel , T. Brezesinski , S. S. Bhattacharya , H. Hahn , B. Breitung , Nat. Commun. 2018, 9, 3400. 30143625 10.1038/s41467-018-05774-5 PMC6109100

[6] N. Qiu , H. Chen , Z. Yang , S. Sun , Y. Wang , Y. Cui , J. Alloys Compd. 2019, 777, 767–774.

[7] Q. Wang , A. Sarkar , Z. Li , Y. Lu , L. Velasco , S. S. Bhattacharya , T. Brezesinski , H. Hahn , B. Breitung , Electrochem. Commun. 2019, 100, 121–125.

[8a] D. Bérardan , S. Franger , D. Dragoe , A. K. Meena , N. Dragoe , Phys. Stat. Sol. RRL 2016, 10, 328–333;

[8b] D. Bérardan , S. Franger , A. K. Meena , N. Dragoe , J. Mater. Chem. A 2016, 4, 9536–9541.

[9a] A. Sarkar , R. Djenadic , N. J. Usharani , K. P. Sanghvi , V. S. K. Chakravadhanula , A. S. Gandhi , H. Hahn , S. S. Bhattacharya , J. Eur. Ceram. Soc. 2017, 37, 747–754;

[9b] J. Zhang , J. Yan , S. Calder , Q. Zheng , M. A. McGuire , D. L. Abernathy , Y. Ren , S. H. Lapidus , K. Page , H. Zheng , J. W. Freeland , J. D. Budai , R. P. Hermann , Chem. Mater. 2019, 31, 3705–3711.

[10a] J. Dabrowa , M. Stygar , A. Mikuła , A. Knapik , K. Mroczka , W. Tejchman , M. Danielewski , M. Martin , Mater. Lett. 2018, 216, 32–36;

[10b] A. Mao , F. Quan , H. Z. Xiang , Z. G. Zhang , K. Kuramoto , A. L. Xia , J. Mol. Struct. 2019, 1194, 11–18.

[11] J. Gild , M. Samiee , J. L. Braun , T. Harrington , H. Vega , P. E. Hopkins , K. Vecchio , J. Luo , J. Eur. Ceram. Soc. 2018, 38, 3578–3584.

[12] A. Sarkar , C. Loho , L. Velasco , T. Thomas , S. S. Bhattacharya , H. Hahn , R. Djenadic , Dalton Trans. 2017, 46, 12167–12176. 28869641 10.1039/c7dt02077e

[13a] A. Sarkar , R. Djenadic , D. Wang , C. Hein , R. Kautenburger , O. Clemens , H. Hahn , J. Eur. Ceram. Soc. 2018, 38, 2318–2327;

[13b] R. Witte , A. Sarkar , R. Kruk , B. Eggert , R. A. Brand , H. Wende , H. Hahn , Phys. Rev. 2019, 3, 034406;

[13c] S. Jiang , T. Hu , J. Gild , N. Zhou , J. Nie , M. Qin , T. Harrington , K. Vecchio , J. Luo , Scripta Mater. 2018, 142, 116–120.

[14] D. A. Vinnik , E. A. Trofimov , V. E. Zhivulin , O. V. Zaitseva , S. A. Gudkova , A. Y. Starikov , D. A. Zherebtsov , A. A. Kirsanova , M. Häßner , R. Niewa , Ceram. Int. 2019, 45, 12942–12948.

[15] A. Sarkar , Q. Wang , A. Schiele , M. R. Chellali , S. S. Bhattacharya , D. Wang , T. Brezesinski , H. Hahn , L. Velasco , B. Breitung , Adv. Mater. 2019, 31, 1806236. 10.1002/adma.201806236 30838717

[16] G. Anand , A. P. Wynn , C. M. Handley , C. L. Freeman , Acta Mater. 2018, 146, 119–125.

[17] J. B. Goodenough , H. Y.-P. Hong , J. Kafalas , Mater. Res. Bull. 1976, 11, 203–220.

[18a] N. Anantharamulu , K. Koteswara Rao , G. Rambabu , B. Vijaya Kumar , V. Radha , M. Vithal , J. Mater. Sci. 2011, 46, 2821–2837;

[18b] M. Guin , F. Tietz , J. Power Sources, 2015, 273, 1056–1064;

[18c] A. Rossbach , F. Tietz , S. Grieshammer , J. Power Sources, 2018, 391, 1–9.

[19] M. E. Brownfield , E. E. Foord , S. J. Sutley , T. Botinelly , Am. Mineral. 1993, 78, 653–656.

[20] Q. Ma , C.-L. Tsai , X.-K. Wei , M. Heggen , F. Tietz , J. Irvine , J. Mater. Chem. A 2019, 7, 7766–7776.

[21] S. Geller , Z. Kristallogr. 1967, 125, 1–47.

[22a] R. Klement , P. Kresse , Z. Anorg. Allg. Chem. 1961, 310, 53–68;

[22b] S. V. Ushakov , A. Navrotsky , J. M. Farmer , L. A. Boatner , J. Mater. Res. 2004, 19, 2165–2175.

[23] R. D. Shannon , Acta Crystallogr. Sect. A 1976, 32, 751–767.

[24a] D. Mazza , M. Lucco-Borlera , S. Ronchetti , Powder Diffr. 1998, 13, 227–231;

[24b] V. Sukhanov , V. I. Pet'kov , D. V. Firsov , V. S. Kurazhkovskaya , E. Yu Borovikova , Russ. J. Inorg. Chem. 2011, 56, 1351–1357;

[24c] V. I. Pet'kov , A. S. Shipilov , M. V. Sukhanov , V. S. Kurazhkovskaya , E. Yu Borovikova , Russ. J. Inorg. Chem. 2014, 59, 1201–1207;

[24d] V. I. Pet'kov , M. V. Sukhanov , A. S. Shipilov , V. S. Kurazhkovskaya , E. Yu Borovikova , I. Yu Pinus , A. B. Yaroslavtsev , Inorg. Mater. 2014, 50, 263–272.

[25a] J. M. Winand , A. Rulmont , P. Tarte , J. Mater. Sci. 1990, 25, 4008–4013;

[25b] F. J. Berry , M. Vithal , Polyhedron 1995, 14, 1113–1115;

[25c] N. Anantharamulu , G. Prasad , M. Vithal , Bull. Mater. Sci. 2008, 31, 133–138.

[26a] J. M. Winand , A. Rulmont , P. Tarte , J. Solid State Chem. 1991, 93, 341–349;

[26b] L.-O. Hagman , P. Kierkegaard , Acta Chem. Scand. 1968, 22, 1822–1832.

[27] Y. Hirata , H. Kitasako , K. Shimada , J. Ceram. Soc. Jpn. 1988, 96, 620–623.

[28] D. O. Savinykh , S. A. Khainakov , A. I. Orlova , S. Garcia-Granda , Russ. J. Inorg. Chem. 2018, 63, 714–724.

[29a] K. S. Nanjundaswamya , A. K. Padhi , J. B. Goodenough , S. Okada , H. Ohtsuka , H. Arai , J. Yamaki , Solid State Ionics 1996, 92,1–10;

[29b] C. W. Mason , I. Gocheva , H. E. Hoster , D. Y. W. Yu , Chem. Commun. 2014, 50, 2249–2251; 10.1039/c3cc47557c 24217427

[29c] S.-C. Chung , J. Ming , L. Lander , J. Lu , A. Yamada , J. Mater. Chem. A 2018, 6, 3919–3925.

[30a] P. R. Slater , C. Greaves , J. Mater. Chem. 1992, 2, 1267–1269;

[30b] P. R. Slater , C. Greaves , J. Solid State Chem. 1993, 107, 12–18;

[30c] P. R. Slater , C. Greaves , J. Mater. Chem. 1994, 4, 1463–1467.

[31] P. Maldonado-Manso , M. C. Martín-Sedeño , S. Bruque , J. Sanz , E. R. Losilla , Solid State Ionics 2007, 178, 43–52.

[32] K. Noi , K. Suzuki , N. Tanibata , A. Hayashi , M. Tatsumisago , J. Am. Ceram. Soc. 2018, 101, 1255–1265.

[33a] H. Chen , H. Tao , Q. Wu , X. Zhao , J. Am. Ceram. Soc. 2013, 96, 801–805;

[33b] W. Ślubowska , K. Kwatek , C. Jastrzębski , J. L. Nowiński , Solid State Ionics 2019, 335, 129–134.

[34] S. Shi , J. Gao , Y. Liu , Y. Zhao , Q. Wu , W. Ju , C. Ouyang , R. Xiao , Chin. Phys. 2016, 25, 018212.

[35a] G. Hautier , Ch. Fischer , A. Jain , T. Mueller , G. Ceder , Chem. Mater. 2010, 22, 3762–3767;

[35b] G. Hautier , Ch. Fischer , V. Ehrlacher , A. Jain , G. Ceder , Inorg. Chem. 2011, 50, 656–663; 10.1021/ic102031h 21142147

[35c] K. Fujimura , A. Seko , Y. Koyama , A. Kuwabara , I. Kishida , K. Shitara , C. A. J. Fisher , H. Moriwake , I. Tanaka , Adv. Energy Mater. 2013, 3, 980–985;

[35d] B. Meredig , A. Agrawal , S. Kirklin , J. E. Saal , J. W. Doak , A. Thompson , K. Zhang , A. Choudhary , C. Wolverton , Phys. Rev. B 2014, 89, 094104.

[36a] B. Lang , B. Ziebarth , C. Elsässer , Chem. Mater. 2015, 27, 5040–5048;

[36b] D. Case , A. J. McSloy , R. Sharpe , S. R. Yeadel , T. Bartlett , J. Cookson , E. Dashjav , F. Tietz , C. M. N. Kumar , P. Goddard , Solid State Ionics 2020, 346, 115192.

