# Supplementary material for: Polyanionic Lattice Modifications Leading to High‐Entropy Sodium Ion Conductors: Mathematical Solution of Accessible Compositions
Source: Chemphyschem. 2020 Aug 26;21(18):2096–103. doi: 10.1002/cphc.202000566 (PMC7540318; doi:10.1002/cphc.202000566)
Supplement: Supplementary file 1 — Supplementary [file CPHC-21-2096-s001.pdf]

ChemPhysChem

Supporting Information

Polyanionic Lattice Modifications Leading to High-Entropy Sodium Ion Conductors: Mathematical Solution of Accessible Compositions

Frank Tietz* and Carsten Fronia

Supporting Information

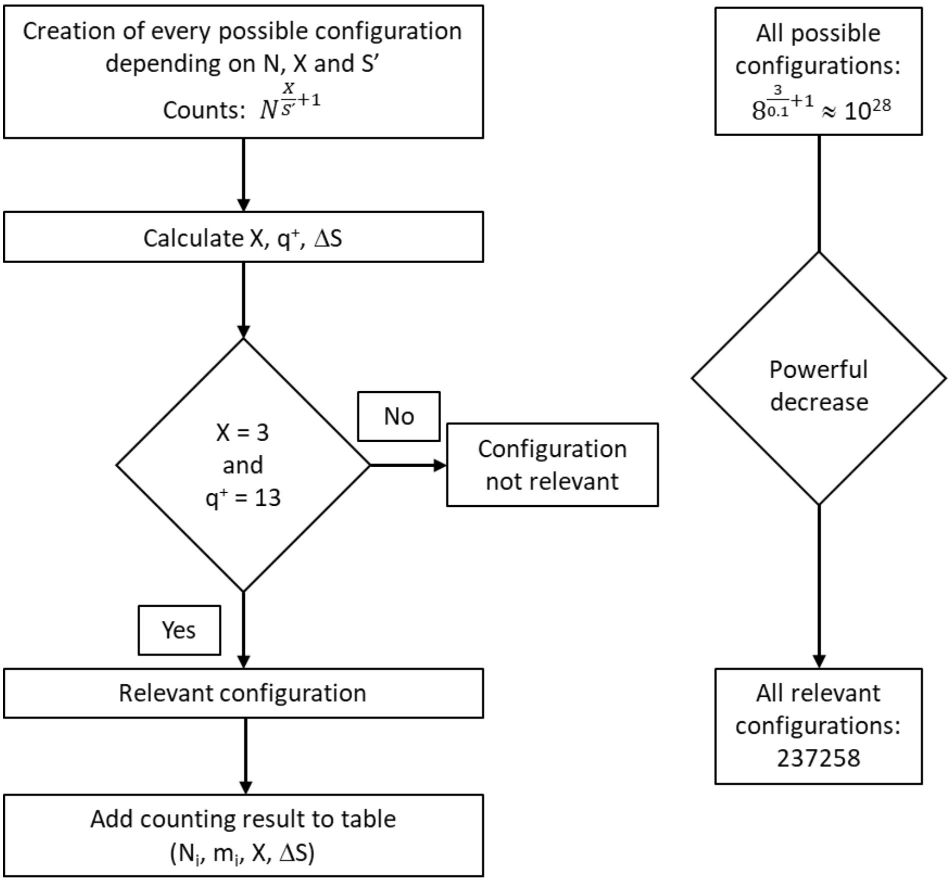

Figure S1. Sequence of computational steps for numerical counting (left) and example of result reduction due to chosen chemical boundary conditions.

Table S1: Statistical overview of the number of compositions with $N \geq 5$ and the involvement of each element for counts with S’ = 0.1. In the first line of each case, the absolute numbers of compositions are given in which the corresponding element appears, the second line corresponds to the percentage of appearances and the third line shows the maximum molar amount ($m_{i,max}$) that can be obtained for each element.

E	Total	B	Al	Si	P	As	Sb	S	Se	Te
B, Al, Si, P, As, Sb, S, Se	232354	210147	210147	199467	188670	188670	188670	177700	177700	-
%		90.44	90.44	85.85	81.20	81.20	81.20	76.48	76.48	-
$m_{i,max}$		1.6	1.6	2.4	1.8	1.8	1.8	1.2	1.2	-
B, Al, Si, P, As, S, Se, Te	180654	165146	165146	155937	146298	146298	-	136971	136971	136971
%		91.42	91.42	86.32	80.98	80.98	-	75.82	75.82	75.82
$m_{i,max}$		1.6	1.6	2.4	1.8	1.8	-	1.2	1.2	1.2
B, Al, Si, P, As, S, Se	43683	39900	39900	38646	37288	37288	-	36046	36046	-
%		91.34	91.34	88.47	85.36	85.36	-	82.52	82.52	-
$m_{i,max}$		1.6	1.6	2.4	1.8	1.8	-	1.2	1.2	-
B, Al, Si, P, As, S	7637	7039	7039	7052	7009	7009	-	7073	-	-
%		92.17	92.17	92.34	91.78	91.78	-	92.61	-	-
$m_{i,max}$		1.5	1.5	2.2	1.8	1.8	-	1.2	-	-
B, Si, P, As, S, Se	3783	3768	-	3581	3391	3391	-	3184	3184	-
%		99.63	-	94.69	89.66	89.66	-	84.19	84.19	-
$m_{i,max}$		1.6	-	2.4	1.7	1.7	-	1.1	1.1	-
B, Si, P, As, S	598	598	-	598	598	598	-	598	-	-
%		100	-	100	100	100	-	100	-	-
$m_{i,max}$		1.5	-	2.2	1.7	1.7	-	1.1	-	-
Si, P, As, S, Se	14	-	-	14	14	14	-	14	14	-
%		-	-	100	100	100	-	100	100	-
$m_{i,max}$		-	-	2.4	0.5	0.5	-	0.3	0.3	-

Table S2: Solution of Equation 1 for 1 < n < 9

n	Fraction	$-\Delta S_{config}/R$
1	1	0
2	0.5	0.693147
3	0.3333	1.098612
4	025	1.386294
5	0.2	1.609438
6	0.1667	1.791759
7	0.1429	1.945910
8	0.125	2.079442
9	0.1111	2.197225

Table S3: Summary of statistical entropy values for various elemental configurations

E = B, Al, Si, P, As, Sb, S, Se						
		Entropy factor $-\Delta S_{config} / R$				
N	No. of all permutations	No. of different values	Mean value	Weighted average value	Minimum value	MaEimum value
5	26208	355	1.3358	1.3840	0.7489	1.6039
6	72749	502	1.5078	1.5569	0.9049	1.7918
7	93531	565	1.6498	1.7082	1.0246	1.9405
8	39866	487	1.7865	1.8387	1.2480	2.0405
sum	232354					
	No. of permutations with $\Delta S_{config} / R \leq -1.609438$					
6	26608	154	1.6794	1.6678	1.6094	1.7918
7	78249	376	1.6498	1.7082	1.6109	1.9405
8	38888	421	1.7865	1.8387	1.6094	2.0405
sum	143745					
E = B, Al, Si, P, As, S, Se, Te						
	No. of all permutations					
5	21469	355	1.3363	1.3743	0.7489	1.6039
6	59963	499	1.5074	1.5475	0.8125	1.7918
7	71161	551	1.6508	1.6962	1.0420	1.9170
8	28061	504	1.7724	1.8186	1.1602	2.0140
sum	180654					
	No. of permutations with $\Delta S_{config} / R \leq -1.609438$					
6	19909	154	1.6794	1.6666	1.6094	1.7918
7	57487	359	1.7464	1.7363	1.6109	1.9170
8	27188	431	1.8187	1.8277	1.6094	2.0140
sum	104584					
E = B, Al, Si, P, As, S, Se						
	No. of all permutations					
5	10216	353	1.3381	1.3834	0.7489	1.6039
6	20235	486	1.4993	1.5606	0.9784	1.7918
7	13232	507	1.6661	1.7132	1.0852	1.9170
sum	43683					
	No. of permutations with $\Delta S_{config} / R \leq -1.609438$					
6	7702	154	1.6794	1.6696	1.6094	1.7918
7	11221	349	1.7479	1.7466	1.6109	1.9170
sum	18923					
E = B, Al, Si, P, As, S						
	No. of all permutations					
5	3601	320	1.3571	1.3907	0.9034	1.6039
6	4036	415	1.5385	1.5740	1.0018	1.7918
sum	7637					
	No. of permutations with $\Delta S_{config} / R \leq -1.609438$					
6	1761	145	1.6792	1.6747	1.6094	1.7918
E = B, Si, P, As, S, Se						
	No. of all permutations					
5	2193	302	1.3336	1.3574	0.7489	1.5722
6	1590	290	1.4912	1.5065	0.9784	1.6859
sum	3783					
	No. of permutations with $\Delta S_{config} / R \leq -1.609438$					
6	264	57	1.6416	1.6365	1.6094	1.6859
